# Flexible Active Peltier Coolers Based on Interconnected Magnetic Nanowire Networks

**DOI:** 10.3390/nano13111735

**Published:** 2023-05-25

**Authors:** Tristan da Câmara Santa Clara Gomes, Nicolas Marchal, Flavio Abreu Araujo, Luc Piraux

**Affiliations:** Institute of Condensed Matter and Nanosciences, Université Catholique de Louvain, Place Croix du Sud 1, 1348 Louvain-la-Neuve, Belgium

**Keywords:** flexible thermoelectrics, active cooling, 3D nanowire networks

## Abstract

Thermoelectric energy conversion based on flexible materials has great potential for applications in the fields of low-power heat harvesting and solid-state cooling. Here, we show that three-dimensional networks of interconnected ferromagnetic metal nanowires embedded in a polymer film are effective flexible materials as active Peltier coolers. Thermocouples based on Co-Fe nanowires exhibit much higher power factors and thermal conductivities near room temperature than other existing flexible thermoelectric systems, with a power factor for Co-Fe nanowire-based thermocouples of about 4.7 mW/K${}^{2}$m at room temperature. The effective thermal conductance of our device can be strongly and rapidly increased by active Peltier-induced heat flow, especially for small temperature differences. Our investigation represents a significant advance in the fabrication of lightweight flexible thermoelectric devices, and it offers great potential for the dynamic thermal management of hot spots on complex surfaces.

## 1. Introduction

Flexible thermoelectric (TE) materials and devices that easily harness the thermal energy of hot surfaces with complex geometries or even the human body for the conversion of heat into electricity offer innovative perspectives in a sustainable development context [1,2]. In particular, flexible TE generators should make it possible to respond to the rapid development of miniature, lightweight and functional portable electronic devices for nomadic products and medical sensors [3,4]. They add their own advantages, linked to their flexibility, lightness and conformability to the main attractions of TE converters, which are the absence of moving mechanical parts and operating noise, high reliability and an almost unlimited lifetime.

Flexible thermoelectrics are generally formed either from fully organic materials or from inorganic/organic hybrid systems, including composites with inorganic nanostructure fillers in a conducting polymer matrix and thin-film inorganic materials deposited on flexible polymer substrates [5,6,7,8,9]. Each of these TE systems requires the development of specific synthesis methods [10,11,12,13,14,15,16,17,18,19,20,21]. These include solution processes for the fabrication of conducting polymers, and physical vapor deposition, spin coating, screen printing, physical mixing and solution mixing for the fabrication of inorganic/organic hybrid systems. Although the thermoelectric properties of conducting polymers and their corresponding nanocomposites have been improved in recent decades, they are still significantly lower than those of bulk thermoelectric materials [5,6,8,9]. In addition, electrical and thermal contact resistances, as well as the mismatch in the thermal expansion coefficients of contacting materials, also affect the performance of such flexible TE devices [22].

Although flexible and lightweight TE devices are expected to generate the same interest in low-power miniaturized refrigeration based on the Peltier effect, it is only very recently that this field of application has received attention [23,24]. Flexible TE devices could enable the development of wearable devices for personalized thermoregulation with the prospect of drastically reducing the volume of heating or cooling by targeting the areas of the human body that require precise thermal doses [23,24,25,26]. In normal operation, Peltier coolers use materials with a high thermoelectric power factor and low thermal conductivity to reduce the reverse heat flow from the hot side. However, the increased development of electronic products, such as computer processors, LEDs and electric batteries, requires that conventional cooling systems be combined with dynamic thermal management to quickly dissipate heat peaks that interfere with the operation of the devices [27,28]. While heat removal from electronic components is usually achieved through high-thermal-conductivity materials that provide passive cooling, thermoelectric cooling has also been considered for dynamic thermal management [29]. Dynamic thermal management is a technique adopted at runtime to minimize hot spots and temperature peaks. Dynamic thermal management is of great interest because of its rapid adaptability to changing environments over time and the energy savings that it offers compared to passive materials. In this particular regime, called active cooling [30,31,32], the Peltier heat flow carried by the electric current is added to the natural flow of heat from the hot to the cold side. As recently demonstrated, materials suitable for the thermal management of electronic hot spots must not only have a high power factor but also high thermal conductivity [33]. Therefore, conventional Peltier TE modules are not suitable for this application due to the low thermal conductivity of their semiconductor components. On the contrary, some transition metals are excellent candidates as thermoelectric materials for active cooling. This is the case for cobalt, which, together with YbAl${}_{3}$ [34], presents the highest room-temperature (RT) thermoelectric power factor among known materials, with a value of around 15 mW/K${}^{2}$m, up to a factor of 10 higher than that of bismuth tellurium [35]. In addition, its thermal conductivity of ∼100 W/Km is very high compared to that of Bi${}_{2}$Te${}_{3}$ (less than 2 W/Km). A new factor of merit, called the effective thermal conductivity, makes it possible to account for the flow of heat transported by materials during active cooling [30,33], i.e., accounting for both passive Fourier heat transport and active Peltier-driven heat flow. The effective thermal conductivity appears as the sum of the “passive” normal thermal conductivity and an “active” thermal conductivity that only starts up when the cooling power supplied by the Peltier module is activated. Using an active cooler made from a rigid bulk-type Co-CePd${}_{3}$ thermoelectric couple, it was found that the effective thermal conductivity of the cooler can exceed 1000 W/Km in its active mode vs. 40 W/Km in the passive mode depending on the hot–cold temperature difference [30]. Although these recently obtained results open up new perspectives in the selection of thermoelectric materials useful for active cooling, hot spots appearing on arbitrarily shaped surfaces require the use of miniaturized, flat and flexible active Peltier coolers. In this context, the easy realization of high-performance flexible thermoelectric modules, with the advantages of reduced weight and size, remains challenging.

In this study, we demonstrate the ability to efficiently cool hot spots on electronic devices using lightweight, flexible Peltier coolers. We achieve this by developing an attractive electrodeposition method of manufacturing thermoelectric modules from ferromagnetic nanowire (NW) networks. Electrochemical synthesis has been shown to be a powerful method for fabricating multicomponent NWs with different metals due to its engineering simplicity, versatility and low cost [36,37,38]. In addition, NW networks have already been proposed as a pathway for efficient thermoelectric devices [39,40]. Herein, NW networks are obtained via simple electroplating within 3D porous polymer membranes so that the nano-constituents active for Peltier cooling are completely embedded and perfectly interconnected within a polymer matrix (see the Experimental Section) [41,42,43,44,45]. In such centimeter-scale NW networks, electrical connectivity is essential to allow charge flow throughout the sample and to preserve the excellent bulk properties. The shapeable nanocomposite films also meet the key requirements for electrical, thermal and mechanical stability.

## 2. Results and Discussion

**Figure 1:**
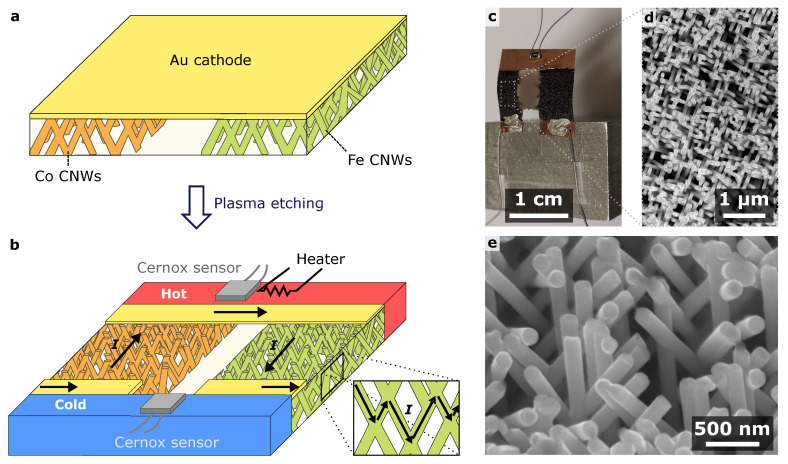
Flexible thermoelectric coolers based on interconnected nanowire networks. (**a**,**b**) Fabrication technique of a thermoelectric device consisting of p- and n-type interconnected metallic nanowire networks (with Fe for p-type and Co for n-type materials). The Co and Fe NW networks, shown in orange and green respectively, are obtained via direct electrodeposition from a Au cathode within a three-dimensional porous polycarbonate membrane (**a**). The thermocouple is made after local removal of the Au electrode via plasma etching (**b**). The temperature at the thermoelectric junction can be raised by means of a resistive heater. Cernox resistance thermometers are used to determine the temperatures of the cold and hot junctions. The inset in (**b**) shows the current path in the nanowire networks. (**c**) Picture of the nanowire-based active cooler corresponding to the schematics in (**b**), showing the flexible and self-supported device. (**d**,**e**) Scanning electron microscopy images of self-supported interconnected Co nanowires with 105 nm diameter showing a 50°-tilted view of the macroscopic nanowire network film (**d**) and the nanowire branched structure at higher magnification (**e**).

Flexible TE modules composed of p-type and n-type legs are fabricated via the successive electrodeposition of arrays of crossed nanowires (CNWs) in a single 22 μm thick polycarbonate (PC) template from a sputtered Au cathode by adapting a previously developed method [41,42,45] (see Figure 1a and the Experimental Section). Here, the TE film devices are made of electrodeposited n-type Co CNW and p-type Fe CNW legs. Next, the Au cathode is removed locally via plasma etching to create the multi-electrode architecture, as shown in Figure 1a,b. In this planar device, the TE elements are connected thermally in parallel and electrically in series, and the current is injected in the macroscopic direction of the film plane taking advantage of the very high degree of electrical connectivity of CNWs, as illustrated in Figure 1b. The device benefits from the mechanical properties of the porous polymer material, which allows for good flexibility and easy handling of the TE films, as shown in Refs. [45,46] (See Supporting Information, Video S1). Figure 1c shows a picture of an example of a nanowire-based active cooler, which highlights that a rigid support is not required to hold the self-supported thermoelectric film device. It should be noted that the dimensions of the device shown in Figure 1c are chosen to better present the characteristics of the system and do not correspond to those of the devices considered in this work, which have much shorter and wider legs to decrease the electrical resistance and increase the performance of the nanowire-based active cooler (see Section 4). The widths of the p and n legs can be selected individually during the successive electrodeposition processes, allowing for thermal and electrical impedance matching, while the lengths of both nanowire legs can be adjusted during the etching process. The fabrication method using track-etch technology allows for thicker nanocomposite films (up to 100 μm) while maintaining flexibility. Note that this system offers much better mechanical properties (in terms of softness and flexibility) than metallic films of similar thicknesses. The polymer matrix also protects the metal NWs from oxidation. In addition, the non-toxicity of the constituent materials makes them wearable devices, whereas toxicity is an issue for many existing flexible thermoelectric devices, apart from conductive polymers [2,3]. The scanning electron microscopy images (see Figure 1d,e) obtained after the complete dissolution of the PC membrane of free-standing CNWs with a 105 nm diameter and a 20% packing density reveal the interconnections between the NWs.

The TE power factors (PF $=S^{2}/\rho$, where ρ is the electrical resistivity, and *S* is the Seebeck coefficient) of the n-type Co CNW and p-type Fe CNW legs are determined separately using previously developed experimental setups for measuring electrical resistance and thermopower as a function of temperature (See Methods and Supporting Information Section S1, Figures S1 and S2). The RT resistivities of the Co and Fe CNW networks are estimated to be 7.1 μΩcm and 12.8 μΩcm, respectively. These values are significantly larger than those of the bulk metals (5.8 μΩcm and 9.8 μΩcm for Co and Fe, respectively). At RT, the resistivity of a bulk metal is fully dominated by large-angle electron–phonon scattering [47]. The residual resistivity due to defect scattering is typically more than a hundred times lower than the resistivity obtained at T= 300 K. On the contrary, in metal NWs, the residual resistivity increases strongly, as evidenced by the reduced residual resistivity ratio (RRR) observed in Co and Fe NWs being close to 4 and 5, respectively (see Supporting Information Section S1). Such an increase in residual resistivity results from an increase in electron scattering caused by structural defects in polycrystalline NWs made using electrodeposition [48] and the surface of the NWs. In particular, it was found for polycrystalline gold NWs that the increase in resistivity due to the external surface scattering of the electrons is not dominant until the diameter is very close to the grain size (40–50 nm) [49]. Therefore, contrary to ultra-small-diameter NWs and thin metal films [50,51], size effects are not very pronounced in our NW system due to the relatively large diameter of the NW (105 nm), so scattering at the grain boundaries is mainly responsible for the increased resistivity of the metal NWs compared to the bulk materials. Furthermore, the temperature dependence and measured Seebeck coefficient values of the NW networks correspond to those of bulk ferromagnets (see See Methods and Supporting Information Section S1, Figure S2). The measured RT values of *S* for the Co and Fe CNW networks are −28 μV/K and +15 μV/K, respectively, values very close to those of the bulk metals (−30 μV/K and +15 μV/K for Co and Fe, respectively).

Consequently, extremely high PF values of ∼11.0 mW/K${}^{2}$m and ∼1.8 mW/K${}^{2}$m at RT are estimated for the Co and Fe CNWs, respectively [45]. These values are only slightly lower than the bulk values, PF ∼15.0 mW/K${}^{2}$m and ∼2.3 mW/K${}^{2}$m for Co and Fe, respectively [35,52,53]. The PF values obtained for these magnetic NWs are even larger than those of the widely used TE material bismuth telluride (in the range of 1–6 mW/K${}^{2}$m [54]) and at least one order of magnitude larger than those reported for flexible thermoelectric films based on optimized conducting polymers and inorganic/organic hybrid systems [6,9,12]. Using these values, the power factor of the Co-Fe nanowire-based thermocouple can be estimated as follows [32,55]:

(1) $${\mathrm{PF}}_{\mathrm{Co}-\mathrm{Fe}}=\frac{{\left(S_{\mathrm{Fe}}-S_{\mathrm{Co}}\right)}^{2}}{{\left(\sqrt{\rho_{\mathrm{Fe}}}+\sqrt{\rho_{\mathrm{Co}}}\right)}^{2}}.$$

At room temperature, the power factor is about 4.7 mW/K${}^{2}$m.

**Figure 2:**
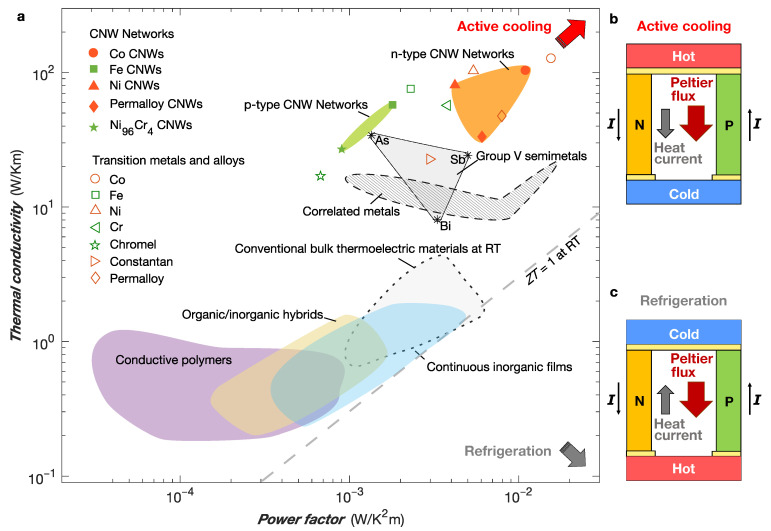
Comparison of the active cooling performance among several flexible thermoelectric materials. (**a**) Thermoelectric power factor PF vs. thermal conductivity κ for flexible TE systems made of n-type (red symbols) and p-type (green symbols) magnetic nanowire networks [45], conductive polymers, organic/inorganic hybrids and continuous inorganic films [1,2,3]. The data for conventional bulk thermoelectric materials near room temperature [64,65,66,67,68,69], correlated metals [34,70,71], group V semimetals [72] and transition metals and alloys [30,35,52,53,73,74,75] are also presented. The gray dashed line shows the case ZT=1 (with Z= PF/κ as the figure of merit), which corresponds to the best power conversion efficiency achievable to date. The most suitable active Peltier coolers are located at the top right of the graph, as indicated by the red arrow. In contrast, the most suitable materials for conventional Peltier refrigeration are located at the bottom right of the graph, as indicated by the gray arrow. (**b**,**c**) Schematic drawings showing the differences between active cooling (**b**) and refrigeration (**c**). In the active cooling mode, Peltier heat flows from the hot side to the cold side, increasing Fourier heat conduction rather than opposing it as in the refrigeration mode.

The complete characterization of the properties of nanostructured thermoelectric materials, including the measurement of thermal conductivity, is generally complex and requires the development of specific integrated measurement devices [56,57,58]. As pointed out above, materials suitable for active cooling must have high thermal conductivity κ, in contrast to traditional thermoelectric materials, which require low thermal conductivity to achieve a high figure of merit Z= PF/κ. Because of the very low thermal conductivity of PC (κ= 0.2 W/Km at RT) and the large packing factor of the NW networks, the contribution of the polymer matrix to heat transport is much smaller than that of the metallic NWs. Although the measurement of the thermal conductivity of isolated NWs and NW networks is very delicate, it has been the subject of several works (for recent reviews, see Refs. [56,57,59]). For metallic NWs, heat is predominantly transported by electrons, and the Wiedemann–Franz law ($\kappa \rho =L_{0}T$, where $L_{0}=$ 2.44 10${}^{-8}$ V${}^{2}$/K${}^{2}$ is the Lorenz number) holds at room temperature to a good approximation, as demonstrated in previous studies on Ni and Ag NWs [60,61,62]. Thus, the thermal conductivity of the Co and Fe NWs can be estimated at RT from their resistivity values, leading to κ∼100 W/Km and κ∼56 W/Km for the Co CNWs and Fe CNWs, respectively. Although these thermal conductivity values are somewhat lower than those of the bulk metals, they are up to two orders of magnitude higher than those of the flexible thermoelectrics developed so far [3,5,63]. It should be noted that the high thermal conductivity of the NW-based nanocomposite system in any direction in the plane of the film, as well as in the direction perpendicular to the film, is directly related to the perfect electrical and thermal connectivity between the NWs along the entire length of the film. In conventional nanocomposite systems consisting of randomly distributed NWs in a polymer matrix, the high thermal contact resistances between the constituents combined with the low thermal conductivity of the polymer lead to significantly lower thermal conductivities. A further comparison of the power factor and thermal conductivity of ferromagnetic CNW networks with those of several flexible and rigid thermoelectric materials is shown in Figure 2a. From these results, it appears that ferromagnetic CNWs are suitable as active TE coolers, as this particular application requires materials with both high PF and high thermal conductivity [30,33]. Based on the thermal conductivity estimates made above, the ZT figure of merit of Co and Fe nanowire networks at room temperature is about 3 × 10${}^{-2}$ and 10${}^{-2}$, respectively. The drawings in Figure 2b,c compare active cooling and refrigeration applications. In the case of active cooling (see Figure 2b), both Peltier flow and Fourier heat conduction are directed from the hot side to the cold side, in contrast to the case of refrigeration (see Figure 2c), where the two heat flows oppose each other.

**Figure 3:**
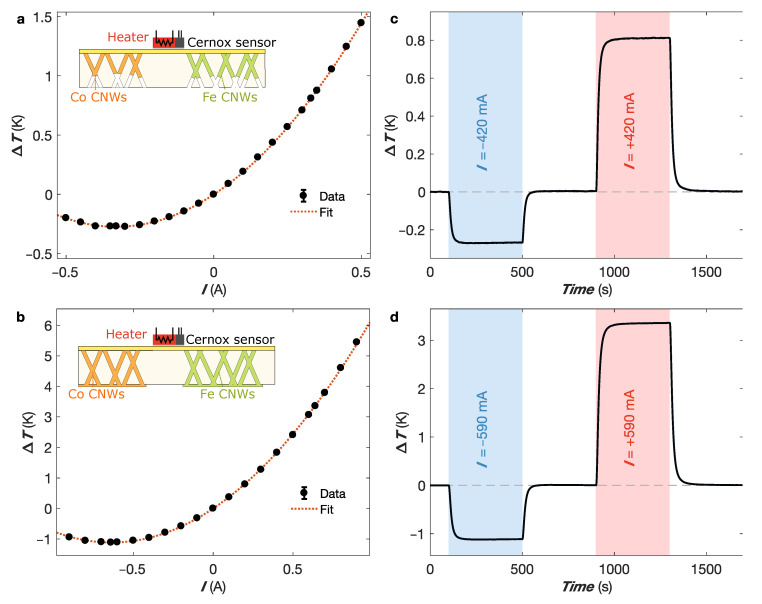
Device characterization as a function of current injection. (**a**,**b**) Measured total temperature changes at the Peltier junction of the NW-based thermocouple versus current intensity applied both forward and reverse for samples 1 (**a**) and 2 (**b**), respectively. The dotted lines provide a fit, including Joule and Peltier contributions to temperature variations. The insets in (**a**,**b**) show schematics of the hot side of thermoelectric coolers consisting of networks of interconnected Co and Fe nanowires of 105 nm in diameter partially filling a porous polymer film (**a**), with the same nanowire constituents completely filling the porous medium with the formation of a thin metallic layer on the surface in (**b**). (**c**,**d**) Temperature versus time traces of the sum of the Joule and Peltier heats relative to a working temperature of 300 K, as recorded using the Cernox sensor. The direct currents of 420 mA for sample 1 (**c**) and of 590 mA for sample 2 (**d**) are applied sequentially forward and reverse in the NW-based thermocouple. Error bars in (**a**,**b**) are smaller than the markers, reflecting the uncertainty of the voltage and temperature measurements, and they are set to two times the standard deviation, gathering 95% of the data variation (See Supporting Information Section S3 for details).

Two CNW thermocouples with different network heights and resistive characteristics are reported here. In sample 1, the PC template is partially filled by the NWs (Figure 3a), thus showing a higher resistance (R= 32.4 mΩ) than sample 2, where the metallic materials completely fill the pores (Figure 3b, R= 23.6 mΩ). The thermocouple legs are approximately 3 mm long, and the widths are set to 22 mm and 40 mm for the Co and Fe CNW legs, respectively, to achieve reasonably good thermal and electrical impedance matching. The two extremities of the thermocouple are connected to the heat sink maintained at RT, while the heated thermocouple junction is physically attached to a Cernox temperature sensor placed in close contact with a resistive heating element, as shown in the insets in Figure 3a,b. The Peltier characteristics of the two samples are first extracted with the resistive heater turned off and by injecting different current values through the sample, which induce both Peltier cooling/heating and Joule heating. The steady-state temperature gradient recorded using the Cernox sensor $\Delta T=T-T_{0}$, where *T* and $T_{0}$ denote the temperatures measured at the thermocouple junction and heat sink, respectively, is reported in Figure 3a,b for the high- and low-resistance samples, respectively. As can be seen, the data (black circles in Figure 3a,b) correspond well to the expected variation due to the Peltier effect contribution (ΔT∝I) and Joule heating contribution ($\Delta T\propto I^{2}$), as shown by the dashed red curves in Figure 3a,b. These combined effects lead to an asymmetric variation in temperature depending on the current direction. The optimal current that minimize ΔT can be estimated from the theoretical expression $I_{\mathrm{opt}}=ST/R$. Considering a sensitivity of 45 μV/K at RT for the Co-Fe thermocouple, this leads to $I_{\mathrm{opt}}=$−420 mA (sample 1) and $I_{\mathrm{opt}}=$−590 mA (sample 2). The experimental results obtained for both samples are in good agreement with these predictions, as shown in Figure 3a,b.

Figure 3c,d show the temperature versus time changes measured using the Cernox sensor for the two samples according to the direction of the DC current supplied to the NW-based Peltier device. After 100 s, a negative current corresponding to the optimal current is injected in the thermocouple for 400 s, leading to a net cooling at the junctions of $\Delta T_{-}=$−0.27 K and $\Delta T_{-}=$−1.12 K for samples 1 and 2, respectively. Then, the current is turned off for 400 s to restore the working temperature of 300 K. Next, changing the direction of the optimal current supplied to the Peltier device leads to temperature increases of $\Delta T_{+}=$+0.81 K and $\Delta T_{+}=$+3.35 K for samples 1 and 2, respectively. The Peltier and Joule contributions at the optimal current, $\Delta T_{P}$ and $\Delta T_{J}$, can be extracted as given by $\Delta T_{P}=\left(\Delta T_{+}-\Delta T_{-}\right)/2=$±0.54 K (±2.23 K) and $\Delta T_{J}=\left(\Delta T_{+}+\Delta T_{-}\right)/2=$ 0.27 K (1.12 K) for the high- (low-) resistance samples, respectively. As can be seen, this sequence applied to the NW-based thermocouple has a fast response and can be switched quickly from heating to cooling.

**Figure 4:**
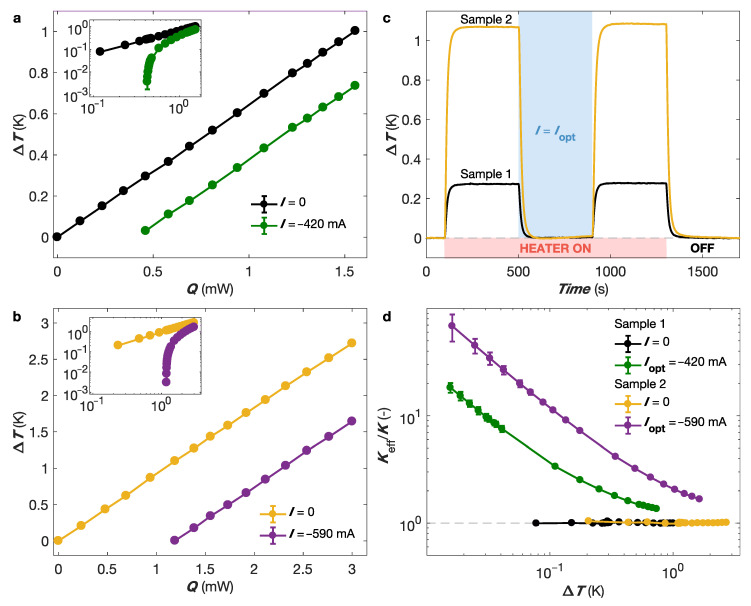
Active cooling of an electronic device. (**a**,**b**) Temperature variations of the Cernox sensor as a function of thermal load when optimal electric currents and zero current are passed through the partially filled (**a**) and completely filled (**b**) Co and Fe nanowire thermocouples. The slopes of the lines provide the thermal conductance. Insets: ΔT vs. *Q* plots on log-log scales. (**c**) The time dependence of temperature changes during successive switching on of the thermal load on the thermoelectric junction and the optimal cooling current of samples 1 (in black, Q= 0.5 mW and $I_{\mathrm{opt}}=$−420 mA) and 2 (in yellow, Q= 1.2 mW and $I_{\mathrm{opt}}=$−590 mA). (**d**) Variation in effective thermal conductance $K_{\mathrm{eff}}$ normalized by the passive-mode thermal conductance *K* with the temperature gradient ΔT for partially filled and completely filled Co and Fe nanowire thermocouples at the optimal switching currents. Error bars in (**a**,**b**,**d**) reflect the uncertainty of the voltage and temperature measurements, and they are set to two times the standard deviation, gathering 95% of the data variation (See Supporting Information Section S3 for details).

Following the measurement scheme shown in Figure 1a and Figure 3a,b, the thermal conductance of the thermoelectric device with a heat sink at ambient temperature is evaluated by heating the thermocouple junction using the heat source provided by the resistive heater and measuring the temperature difference $\Delta T=T-T_{0}$ after establishing a steady-state temperature distribution. For the passive cooling (I= 0) regime, the proportional relationship between the known power input *Q* and the resulting temperature drop is shown in Figure 4a,b for both samples. The same measurements are performed in the active cooling regime using the optimum currents for sample 1 ($I_{\mathrm{opt}}=$−420 mA) and sample 2 ($I_{\mathrm{opt}}=$−590 mA). As expected, the active Peltier effect leads to smaller ΔT values for a given power dissipation in the heater than the passive cooling state. In contrast to the passive mode, the relationship between *Q* and ΔT in the active mode is no longer linear in particular for small temperature differences, indicating an increase in thermal conductance, as given by Q/ΔT (see insets in Figure 4a,b). Figure 4c shows the temperature versus time traces obtained during the successive switching on of the thermal load on the thermoelectric junction and the optimal cooling currents of samples 1 (in black) and 2 (in yellow). For sample 1, the temperature increase resulting from a power dissipation P= 0.5 mW in the resistive heater can be completely compensated by the NW-based TE cooler operating at its optimum current of −420 mA (Figure 4c in black). Similarly, for sample 2, a ΔT of 1.1 K resulting from a thermal load of 1.2 mW in the heater can be canceled out by injecting an optimal Peltier current of −590 mA (Figure 4c in yellow).

At the optimal current $I_{\mathrm{opt}}$, the ratio between the effective thermal conductance $K_{\mathrm{eff}}$ (including both the passive cooling and active Peltier contributions—see Supporting Information Section S2) and the thermal conductance of the thermocouple is given by [30,33]

(2) $$\frac{K_{\mathrm{eff}}}{K}=\left[\frac{1}{2}\frac{{\left(S_{p}-S_{n}\right)}^{2}T_{H}^{2}}{KR\Delta T}+1\right],$$

where $S_{p}$ and $S_{n}$ are the respective Seebeck coefficients for the p and n legs, *R* is the electrical resistance, and $T_{H}$ is the hot-side temperature. This expression indicates that $K_{\mathrm{eff}}/K$ increases when the temperature difference is low, in agreement with the results reported in Figure 4d for samples 1 and 2. As an example, for ΔT= 0.1 K, the gain on the thermal conductance due to active cooling reaches factors 4 and 12 for samples 1 and 2, respectively. It should be noted that a decrease in contact resistance, which can hardly be made negligible compared to that of the conductive film formed by the NW array (see Section 4), should lead to even better performance. Another limiting factor is radiation losses, which are not evaluated in this study.

For metallic legs, Equation (2) can be simplified as follows:

(3) $$\frac{K_{\mathrm{eff}}}{K}=\left[\frac{1}{2}\frac{{\left(S_{p}-S_{n}\right)}^{2}T_{H}}{L_{0}\Delta T}+1\right],$$

where $L_{0}$ is the Lorenz number, equal to the ratio KR/T in the Wiedemann–Franz law. Equation (3) makes it easy to estimate, for a given metallic thermocouple, the maximum thermal conductance gain at any working temperature for various values of ΔT. It should be noted that Equation (3) does not take into account additional heat losses due to radiation or the contribution of contact resistances that reduce the $K_{\mathrm{eff}}/K$ ratio, as observed in our Co-Fe CNW thermocouples (see Figure 4d). For a Co-Fe thermocouple, this leads to $K_{\mathrm{eff}}/K\approx$ 13 for ΔT= 1 K at RT. Therefore, the effective thermal conductivities of the Co and Fe legs with bulk values of ∼125 W/Km and ∼80 W/Km, respectively, both exceed 1000 W/Km for ΔT= 1 K, which corresponds to the values of the best thermal conductors at RT. In addition, a larger improvement can be obtained when the temperature of the hot spot is higher than RT. From Equation (3), it is also seen that active Peltier cooling removes more heat than passive cooling if $\Delta T<{\left(S_{p}-S_{n}\right)}^{2}T_{H}/2L_{0}$, which corresponds to ΔT< 12.4 K for a Co-Fe thermocouple at ambient temperature. Furthermore, as the thermal conductivity of Cu is about four times higher than that of Co NWs, it can be seen from Equation (3) that, under ideal conditions and for a ΔT lower than about 3.8 K, the Co-Fe NW thermocouple has a better cooling capacity in active mode than passive heat transfer via Cu. Moreover, it should be noted that the main advantages of active Peltier cooling are the dynamic and controllable aspects, which allow for dynamic heat removal for precise temperature control. In a regime of rapid temperature increases in small amounts (i.e., small ΔT), the active Peltier cooler can effectively increase the effective thermal conductance of the cooling system, which can be precisely controlled by adjusting the electric current flowing through the thermocouple. Therefore, the large improvement in effective thermal conductance in our flexible Co-Fe NW-based devices holds promise for the accurate and fast-response temperature management of hot spots in electronic devices. Furthermore, by simply reversing the polarity of the current, the effective conductance can be significantly reduced, allowing for the easy realization of flexible thermal switches, which have great potential for heat management; thermal logic devices; and magnetocaloric/electrocaloric coolers [76,77].

## 3. Conclusions

In this paper, we propose a simple, versatile and reliable fabrication technique for lightweight, non-toxic, flexible and shapeable TE devices consisting of interconnected nanowire arrays embedded in 22 μm thick polymer films. The combination of a high power factor and high thermal conductivity in ferromagnetic nanowires provides flexible active Peltier coolers with significantly better performance than other existing flexible TE systems. A power factor of about 4.7 mW/K${}^{2}$m was obtained at room temperature for Co-Fe nanowire-based thermocouples, providing the highest value reported to date for flexible TE films. We found that the ratio of the effective thermal conductance of Co-Fe nanowire-based thermocouples in the active mode to the thermal conductance in the passive mode increases sharply as the hot–cold temperature difference decreases. The process of integrating electrochemically produced nanowire thermocouples into active TE coolers is simple to implement. The size of the planar device and thermocouples, as well as the number of modules, can be adjusted and optimized to cool hot spots on surfaces with complex geometries. Another interesting prospect of 3D nanowire networks is the possibility to fabricate multilayer nanowires to achieve magnetic control of the thermoelectric properties [41,46]. It should be noted that a route for the fabrication of vertical thermocouples based on CNWs has very recently been proposed [46]. This architecture, which is closer to that generally found in usual thermoelectric devices and offers excellent prospects for flexible active coolers, requires further optimization to limit contact resistance issues. These results represent a significant advance in the design of flexible TE device assemblies and offer real prospects for the thermal management of electronic hot spots.

## 4. Experimental Section

### 4.1. Fabrication of Flexible Nanowire-Based TE Devices

Polycarbonate (PC) porous membranes with interconnected pores were fabricated by exposing a 22 μm thick PC film to a two-step irradiation process [78,79]. The topology of the membranes was defined by exposing the film to an initial irradiation step at two fixed angles of −25° and +25° with respect to the normal axis of the film plane. After rotating the PC film in the plane by 90°, the second irradiation step took place at the same fixed angular irradiation flux to finally form a 3D nanochannel network. Then, latent tracks were chemically etched following a previously reported protocol [80] to obtain 3D porous membranes with pores 105 nm in diameter and a volumetric porosity of 20%. Next, the PC templates were coated on one side using an e-beam evaporator with a metallic Cr (3 nm)/Au (700 nm) bilayer to serve as the cathode during electrochemical deposition.

Flexible NW-based TE devices were manufactured via the successive electroplating of n-type Co NW legs and p-type Fe NW legs. Electrodeposition was performed at RT in the potentiostatic mode using a Ag/AgCl reference electrode and a Pt counter electrode. The electrodeposition of Fe and Co CNWs into interconnected pore PC templates was carried out at the respective constant potentials of −1.20 V and −0.95 V using the following electrolytes: 0.5 M FeSO${}_{4}$· 7 H${}_{2}$O + 0.5 M H${}_{3}$BO${}_{3}$ at pH 2, and 0.5 M CoSO${}_{4}$· 7 H${}_{2}$O + 0.5 M H${}_{3}$BO${}_{3}$ at pH 3.6.

The dimensions of the p and n NW legs were 40 mm × 3 mm and 22 mm × 3 mm in area, respectively, and they were 22 μm thick when pore filling was complete. After the electroplating process, a single etching step carried out using plasma etching through a mechanical mask allowed for the local removal of the surface gold layer to finalize the design of the planar TE module. At the end of this etching step, only metallic segments of the continuous gold layer remained, which allowed for the thermoelectric legs p and n to be alternately connected in series.

### 4.2. Electrical and Thermoelectric Measurements

Thermoelectric and resistivity measurements of individual Co NW and Fe NW networks were performed as a function of temperature using homemade setups. For conducting electrical and thermoelectric transport measurements, the cathode was locally removed via plasma etching to create a two-probe design suitable for electric measurements (see Supporting Information Section S1, Figure S1). In this configuration, the current was directly injected to the branched CNW structure of the film samples from unetched sections of the metallic cathode, where the electrical contacts were directly made using Ag paint, and it passed through the NW network thanks to the high degree of electrical connectivity of the CNWs. The typical resistance values of the prepared specimens were in the range of a few tens of ohms. For each sample, the input power was kept below 0.1 μW to avoid self-heating, and the resistance was measured within its ohmic resistance range with a resolution of one part in 10${}^{5}$. The RT resistivities of the Co and Fe NW networks were estimated from low-temperature resistance measurements assuming that the Matthiesen’s rule holds. In this case, the resistivity at RT is given by $\rho_{\mathrm{NWs}}^{\mathrm{RT}}=\rho_{\mathrm{FM}}^{\mathrm{RT}}+\rho_{\mathrm{NWs}}^{0}$, where $\rho_{\mathrm{FM}}^{\mathrm{RT}}$ is the resistivity of the FM that composes the NWs at RT due to thermally excited scatterings, and $\rho_{\mathrm{NWs}}^{0}$ is the residual resistivity of the NWs due to impurities, and surface and grain-boundary scatterings. For an NW diameter that is not too small (ϕ≤ 40 nm), the thermally induced scattering effects are independent from the sample dimensions, nanostructuration and defect concentration [81]. Therefore, $\rho_{\mathrm{FM}}^{\mathrm{RT}}$ can be taken as the ideal resistivity value at RT reported for bulk materials; i.e., $\rho_{\mathrm{Co}}^{\mathrm{RT}}=$ 5.8 μΩcm and $\rho_{\mathrm{Fe}}^{\mathrm{RT}}=$ 9.8 μΩcm. Moreover, the resistivity of the NW networks at T= 10 K can be approximated to $\rho^{10}\;K_{\mathrm{NWs}}∼\rho_{\mathrm{NWs}}^{0}$. Finally, using the measured residual resistivity ratio RRR $=R_{\mathrm{NWs}}^{\mathrm{RT}}/R^{10}\;K_{\mathrm{NWs}}∼\left(\rho_{\mathrm{FM}}^{\mathrm{RT}}+\rho_{\mathrm{NWs}}^{0}\right)/\rho_{\mathrm{NWs}}^{0}$ (see Supporting Information Section S1, Figure S2a), the RT resistivity of the NWs can be estimated as $\rho_{\mathrm{NWs}}^{\mathrm{RT}}∼\rho_{\mathrm{FM}}^{\mathrm{RT}}\mathrm{RRR}/\left(\mathrm{RRR}-1\right)$. The resistivity values obtained for the CNW systems ($\rho_{\mathrm{Co}}^{\mathrm{RT}}=$ 7.1 μΩcm and $\rho_{\mathrm{Fe}}^{\mathrm{RT}}=$ 12.8 μΩcm) were slightly larger than those obtained for the bulk materials, as expected for electrodeposited nanostructured materials.

The thermoelectric power was measured by attaching one end of the sample to a copper sample holder using silver paint and a resistive heater to the other end (see Supporting Information Section S1, Figure S1). The voltage leads were made of thin Chromel P wires, and the contribution of the leads to the measured thermoelectric power was subtracted using the recommended values for the absolute thermopower of Chromel P. The temperature gradient was monitored with a small-diameter type E differential thermocouple. A typical temperature difference of 1 K was used in the measurements. Electrical and thermoelectric measurements were performed under vacuum. The temperature of the samples can be varied from 10 to 320 K.

Active Peltier cooling experiments were conducted at room temperature on two different NW-based thermocouples made of interconnected Co and Fe NWs 105 nm in diameter. In the first NW-based thermocouple, the NW network partially filled the 22 μm thick porous polymer film, with a resistance of 32.4 mΩ. In the second specimen, the same nanowire constituents completely filled the porous medium with the formation of a thin metallic layer on the surface, thus leading to a lower resistance of 23.6 mΩ. Through independent characterization tests, the electrical contact resistance was estimated at ∼6 ± 2 mΩ, thus being significantly smaller, although not negligible, than the resistance of the two samples investigated in this study. The relatively low contact resistance can be attributed to the large surface area of the electrode used as a cathode for direct nanowire growth via electrodeposition combined with the interconnected structure of the nanowire network and its high packing factor (∼20%). The heat absorbed or released at the thermoelectric junction, whose temperature can be raised by means of a resistive heater, was measured using a small Cernox thin-film resistance sensor (<3 mg, 1 mm${}^{2}$; Cernox 1010, Lake Shore Cryotronics Inc., USA) attached at the junctions between the NW networks. The temperature resolution of this highly sensitive thermometer is about 1 mK, which enables the detection of Peltier-effect-based heating or cooling, while a DC electrical current was applied sequentially forward and reverse in the NW-based thermocouples.

## Data Availability

The data presented in this study are available on request from the corresponding author.

## Supplementary Materials

The following supporting information can be downloaded at: https://www.mdpi.com/article/10.3390/nano13111735/s1, Figure S1: Device configuration for measurements of the resistance and the Seebeck coefficient of the metallic NW networks. a, Schematic of 3D interconnected nanowire network film grown by electrodeposition from a Au cathode into a 22 µm thick polycarbonate template with crossed-nanopores. b, Two-probe electrodes design obtained by local etching of the Au cathode. c, The voltage differential ΔV induced by the injected current *I* between the two metallic electrodes is measured while the two electrodes are maintained at an identical and constant temperature. d, Heat flow is generated by a resistive element at one electrode while the other electrode is maintained at desired temperature. The temperature difference ΔT between the two metallic electrodes is measured by a thermocouple while thermoelectric voltage ΔV settles. Figure S2: Temperature variation of the electrical resistance and Seebeck coefficient of the Co and Fe CNW networks. a, Measured resistance vs. temperature curves for the interconnected Co and Fe NW networks, 105 nm in diameter, 10 mm × 2.5 mm × 0.022 mm (thickness) in sizes. b, Measured S(T) curves obtained on the same samples. The data are compared with the bulk values indicated by the dashed lines. Error bars are smaller than the markers, reflecting the uncertainty of the voltage and temperature measurements and set to two times the standard deviation, gathering 95% of the data variation. Video S1: Movie showing the flexible thermoelectric active cooler composed of three p-n modules. The electrical resistance of the nanowire nanocomposite remains unchanged when subjected to twisting movements. References [82,83] are cited in the supplementary materials.
