# Supplementary material for: Flexible Active Peltier Coolers Based on Interconnected Magnetic Nanowire Networks
Source: Nanomaterials (Basel). 2023 May 25;13(11):1735. doi: 10.3390/nano13111735 (PMC10254881; doi:10.3390/nano13111735)
Supplement: Supplementary file 1 [file nanomaterials-13-01735-s001.zip › SupplementaryInformation.pdf]

Supplemental material for:

Flexible active Peltier coolers based on interconnected magnetic nanowire networks

*Tristan da Câmara Santa Clara Gomes, Nicolas Marchal, Flavio Abreu Araujo and Luc Piraux**

Institute of Condensed Matter and Nanosciences, Université catholique de Louvain, Place Croix du Sud 1, Louvain-la-Neuve, 1348, Belgium

S1 Electric and thermoelectric measurements of nanowire networks

To perform electric and thermoelectric measurements on 3D CNW networks, the Au layer that serves as cathode during the electrodeposition process was locally etched to allow two-probe measurement, as shown in Figures S1a-b. For these preliminary characterization measurements of the nanowire arrays, the results obtained in a 2-probe configuration are identical to those obtained using a 4-probe configuration. This is due to the fact that the CNW samples used for these measurements only partially filled the porous templates and have high length/cross section ratios leading to high sample resistances relative to the contact resistance. The length, width and thickness of such interconnected NW networks are 10 mm, 2.5 mm, and 0.005 mm. Therefore, for electric measurements, a current I is injected through the sample from the electrode probe while the voltage differential ΔV is measured, which allows to obtain the NW resistance as $R = \Delta V/I$, while the two electrodes are maintained at identical temperature (see Figure S1c). For thermoelectric measurements, a resistive heater is connected to one electrode while the other is connected to a heat sink to induce a temperature differential ΔT at the CNW network edges, while the voltage differential ΔV is measured. The CNW network Seebeck coefficient S is obtained by removing the contribution of the Chromel P wire leads $S_{\text{Crp}}(T_{\text{av}})$ as $S = \zeta\Delta V/\Delta T - S_{\text{Crp}}(T_{\text{av}})$, where $T_{\text{av}} = (T_{\text{heatsink}} + 0.5\Delta T)$, and ζ is a correction factor estimated to 0.88 related to relative contact positions for the measurements of ΔV and ΔT [1].

Figure S2a shows the measured resistance R as a function of temperature on 3D CNW networks made of Co and Fe, normalized by the resistance at $T = 300$ K, showing a linear decrease with decreasing temperature until a plateau is reached for $R/R_{300\,\text{K}} \approx 0.2$ (i.e. a residual resistivity ratio RRR of about 5, with RRR $\approx R_{300\,\text{K}}/R_{10\,\text{K}}$). From this, the resistivity of the CNW networks can be estimated assuming that the Matthiesen's rule holds for the different CNW networks. In that case, the resistivity at RT is given by $\rho^{\text{RT}}_{\text{NWs}} = \rho^{\text{RT}}_{\text{FM}} + \rho^{0}_{\text{NWs}}$, where $\rho^{\text{RT}}_{\text{FM}}$ is the resistivity of the ferromagnetic material (FM) that composes the CNWs at RT due thermally excited scatterings and ρ^{0}_{NWs} is the residual resistivity of the CNWs due to impurities along with surface scattering within the CNW network and internal grain-boundary scattering. For NW diameter not too small ($\phi \geq 40$ nm), the thermally induced scattering effects are independent on the sample dimensions, nanostructuration and defect concentration [2]. Therefore, $\rho^{\text{RT}}_{\text{FM}}$ can be taken as the ideal resistivity values at RT reported for bulk materials in the literature. As seen in Figure S2a, the plateau is already reached at 10 K so that the resistivity at 10 K of the CNW networks can be approximated to ρ^{0}_{NWs}. This yields RRR $\approx (\rho^{\text{RT}}_{\text{FM}} + \rho^{0}_{\text{NWs}})/\rho^{0}_{\text{NWs}}$ so that the RT resistivity of the NWs can be estimated as $\rho^{\text{RT}}_{\text{NWs}} \sim \rho^{\text{RT}}_{\text{FM}}$ RRR/(RRR $-$ 1). Taking $\rho^{\text{RT}}_{\text{Co}} = 5.8$ $\mu\Omega$cm and $\rho^{\text{RT}}_{\text{Fe}} = 9.8$ $\mu\Omega$cm [3] and the measured RRR of 5.4 and 4.3 for Co and Fe CNW networks, respectively gives RT resistivty for the Co and Fe CNW networks of $\rho^{\text{RT}}_{\text{Co NWs}} \approx 7.1$ $\mu\Omega$cm and $\rho^{\text{RT}}_{\text{Fe NWs}}$ 12.8 $\mu\Omega$cm, respectively.

Figure S2b shows the measured Seebeck coefficient S as a function of temperature on 3D CNW

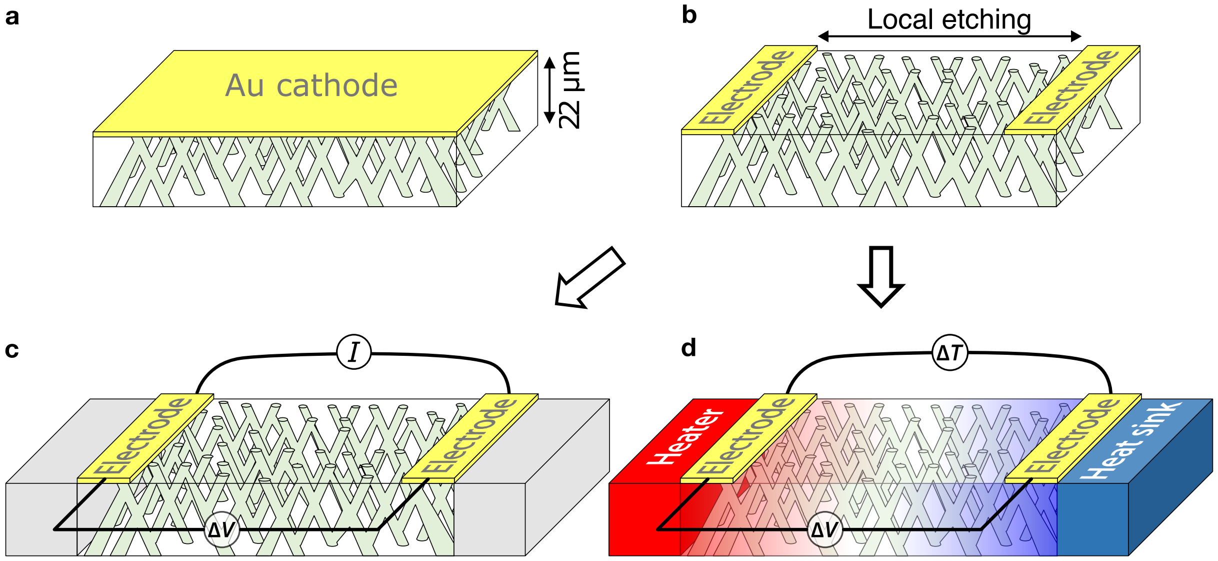

Figure S1: Device configuration for measurements of the resistance and the Seebeck coefficient of the metallic NW networks. a, Schematic of 3D interconnected nanowire network film grown by electrodeposition from a Au cathode into a 22 μm thick polycarbonate template with crossed-nanopores. b, Two-probe electrodes design obtained by local etching of the Au cathode. c, The voltage differential ΔV induced by the injected current I between the two metallic electrodes is measured while the two electrodes are maintained at an identical and constant temperature. d, Heat flow is generated by a resistive element at one electrode while the other electrode is maintained at desired temperature. The temperature difference ΔT between the two metallic electrodes is measured by a thermocouple while thermoelectric voltage ΔV settles.

networks made of Co and Fe (dots) compared to the bulk Co [4] and Fe [5] values. As seen, the data measured on our 3D CNW network correspond well to that of the expected bulk values. The measured RT values of S for the Co and Fe CNW networks were respectively -28 μV/K and $+15$ μV/K.

S2 Theoretical calculation of the effective conductance.

The cooling power Q_c of a thermoelectric active cooler consisting of a p-type leg and a n-type leg is

$$Q_\mathrm{c} = (S_\mathrm{p} - S_\mathrm{n})IT_\mathrm{H} + K(T_\mathrm{H} - T_\mathrm{C}) - 1/2RI^2, \tag{S1}$$

where S_p and S_n are the respective Seebeck coefficients for the p and n legs, I is the electric current, T_H and T_C are the hot-side and cold-side temperatures, respectively, and K and R are the thermal conductance and electrical resistance of the thermocouple.

The maximum active cooling rate achieved at the optimal current $I_\mathrm{opt} = ((S_\mathrm{p} - S_\mathrm{n})T_\mathrm{H})/R$ is given as

$$Q_\mathrm{c,\,opt} = \frac{1}{2}\frac{(S_\mathrm{p} - S_\mathrm{n})^2 T_\mathrm{H}^2}{R} + K(T_\mathrm{H} - T_\mathrm{C}). \tag{S2}$$

In the ideal case where the dimensions of the n and p metallic legs are adjusted to match their electrical and thermal conductance to each other and assuming negligible contact resistances and radiative losses, an effective thermal conductance K_eff of the thermocouple including both the passive cooling contribution and the active Peltier cooling contribution can be defined from Equation S2 as follows

$$K_\mathrm{eff} = \frac{1}{2}\frac{(S_\mathrm{p} - S_\mathrm{n})^2 T_\mathrm{H}^2}{R\Delta T} + K, \tag{S3}$$

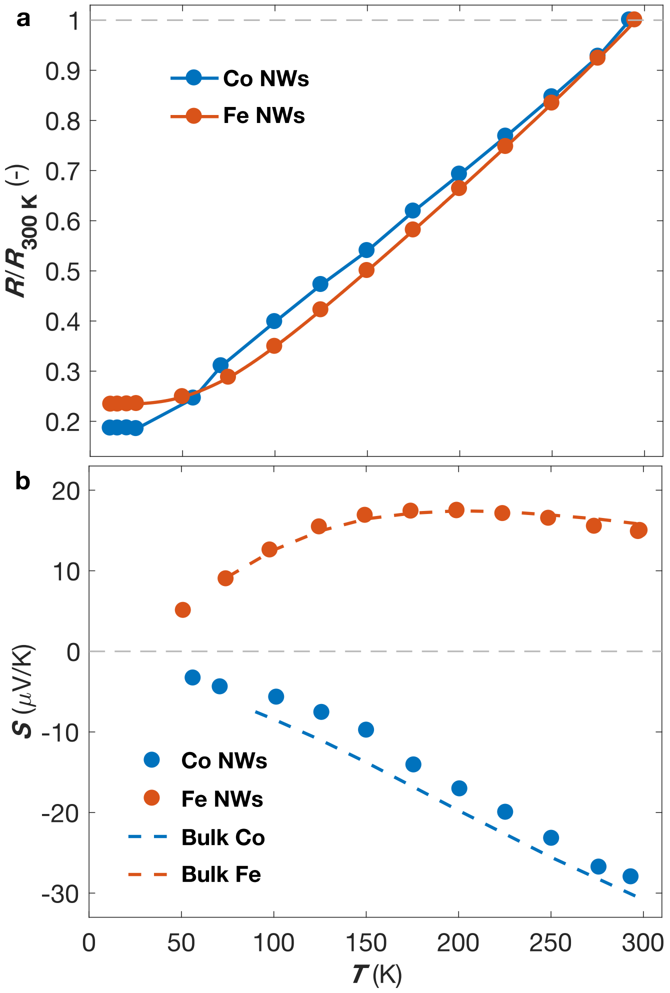

Figure S2: Temperature variation of the electrical resistance and Seebeck coefficient of the Co and Fe CNW networks. a, Measured resistance vs. temperature curves for the interconnected Co and Fe NW networks, 105 nm in diameter, 10 mm x 2.5 mm x 0.022 mm (thickness) in sizes. b, Measured $S(T)$ curves obtained on the same samples. The data are compared with the bulk values indicated by the dashed lines. Error bars are smaller than the markers, reflecting the uncertainty of the voltage and temperature measurements and set to two times the standard deviation, gathering 95% of the data variation.

with $\Delta T = T_{\mathrm{H}} - T_{\mathrm{C}}$.

As shown in ref. [6], Equation S3 applies to a thermoelectric thermocouple, but it could be extended to a single thermoelectric leg by writing the equations S1 and S2 in the form of heat flux (i.e. in units of W/m^2) so that geometrical dependences are removed. In this case, one can write

$$\kappa_{\mathrm{eff}} = \kappa + \frac{1}{2}\frac{\mathrm{PF}T_{\mathrm{H}}^2}{\Delta T}, \tag{S4}$$

with PF the power factor. It is noted that power-factor multiplied by temperature has the same unit as the thermal conductivity. From Equation S4 it appears that the effective thermal conductivity is the sum of the usual passive thermal conductivity and the so-called active thermal conductivity term associated to the Peltier-induced active heat flow.

S3 Experimental measurement uncertainty evaluation.

The computation of the error bars is subject to the standard combination of the root-mean-square errors of the temperatures measured. Once the system reaches the steady-state, 100 temperature

measurements are taken to estimate the standard mean deviations (σ) of each measurements.

Before each measurement, 100 measurements of T_0 are made to get $\sigma(T_0)$. For each measurements, the application of a current to the heater or in the samples takes 400 s, where 300 s are given to the system to reach steady-state and 100 s with 1 measurement of the temperature per second are made to estimate the temperature reached (mean value over the 100 measures) and the standard deviation $\sigma(T)$. The error, or uncertainty, considered for these variables is set to 2 times σ (gathering 95% of the data variation) from the mean values. The rigorous combination of the different errors gives rise to the uncertainty of the parameters shown in our graphs. The error over ΔT is then computed as:

$$\Delta T_{\text{err}} = \sqrt{T_{\text{err}}^2 + T_{0,\,\text{err}}^2}, \tag{S5}$$

where $T_{0,\,\text{err}}$ and T_{err} are the errors of the base temperature T_0 and the temperature after turning on the heater.

Moreover, each action (applying a current to the heater or the sample) are reversed in the same condition to verify the previous temperature. For instance, for Peltier measurements (Figures 2c-d), 100 measurements are used to obtain the base temperature T_0 and its standard variation $\sigma(T_0)$, then a negative current is applied during 400 s (one measurement of T per second) with the last 100 measurements used to obtain the steady state temperarure induced by Joule and Peltier effect T_- and its standard variation $\sigma(T_-)$. After that, the current is removed during 400 s, with the last 100 measurements used to measure again T_0 and $\sigma(T_0)$. Then, a positive current is applied during 400 s with the last 100 measurements used to obtain T_+ and $\sigma(T_+)$, before removing it again to measure again T_0 and $\sigma(T_0)$. This allows to verify that no temperature shift are present during the complete measurements.

References

[1] N. Marchal, T. da Câmara Santa Clara Gomes, F. Abreu Araujo, L. Piraux, *Nanoscale Research Letters* **2020**, *15*, 1 137.

[2] M. V. Kamalakar, A. K. Raychaudhuri, *Phys. Rev. B* **2009**, *79* 205417.

[3] G. T. Meaden, *Electrical resistance of metals*, Springer, **1965**.

[4] M. J. Laubitz, T. Matsumura, *Canadian Journal of Physics* **1973**, *51*, 12 1247.

[5] F. J. Blatt, *Canadian Journal of Physics* **1972**, *50*, 22 2836.

[6] M. Adams, M. Verosky, M. Zebarjadi, J. Heremans, *Phys. Rev. Applied* **2019**, *11* 054008.
